# Safety, Immunogenicity, and Effectiveness of Chinese-Made COVID-19 Vaccines in the Real World: An Interim Report of a Living Systematic Review

**DOI:** 10.3390/vaccines12070781

**Published:** 2024-07-16

**Authors:** Yangyang Qi, Hui Zheng, Jinxia Wang, Yani Chen, Xu Guo, Zheng Li, Wei Zhang, Jiajia Zhou, Songmei Wang, Boyi Lin, Lin Zhang, Tingting Yan, John Clemens, Jielai Xia, Zhijie An, Zundong Yin, Xuanyi Wang, Zijian Feng

**Affiliations:** 1 Shanghai Institute of Infectious Disease and Biosecurity, Fudan University, Shanghai 200032, China; yyqi22@m.fudan.edu.cn (Y.Q.); zhengli22@m.fudan.edu.cn (Z.L.); 2 Key Laboratory of Medical Molecular Virology of MoE & MoH and Institutes of Biomedical Sciences, Shanghai Medical College, Fudan University, Shanghai 200032, China; 3 National Immunization Program, Chinese Center for Disease Control and Prevention, Beijing 102206, China; zhenghui@chinacdc.cn (H.Z.); chenyn@chinacdc.cn (Y.C.); guoxu@chinacdc.cn (X.G.); zhoujj@chinacdc.cn (J.Z.); linby@chinacdc.cn (B.L.); zhangl@chinacdc.cn (L.Z.); yantt@chinacdc.cn (T.Y.); anzj@chinacdc.cn (Z.A.); yinzd@chinacdc.cn (Z.Y.); 4 Clinical Research Unit, Shanghai Children’s Hospital, School of Medicine, Shanghai Jiao Tong University, Shanghai 200240, China; wangjinxia@shchildren.com.cn; 5 Medical Library, Fudan University Library, Fudan University, Shanghai 200032, China; weizhang@shmu.edu.cn; 6 Laboratory of Molecular Biology, Training Center of Medical Experiments, School of Basic Medical Sciences, Fudan University, Shanghai 200032, China; smwang2@fudan.edu.cn; 7 International Vaccine Institute, Seoul 08826, Republic of Korea; john.clemens@ivi.int; 8 Xijing Hospital, Air Force Medical University, Xi’an 710032, China; xiajielai@fmmu.edu.cn; 9 Children’s Hospital, Fudan University, Shanghai 200032, China; 10 Chinese Preventive Medicine Association, Beijing 100009, China

**Keywords:** effectiveness, safety, inactivated vaccines, COVID-19, meta-analysis, China

## Abstract

**Background:** Several COVID-19 vaccines were developed and approved in China. Of these, the BIBB-CorV and CoronaVac inactivated whole-virion vaccines were widely distributed in China and developing countries. However, the performance of the two vaccines in the real world has not been summarized. **Methods:** A living systematic review based on findings from ongoing post-licensure studies was conducted, applying standardized algorithms. Articles published between 1 May 2020 and 31 May 2022 in English and Chinese were searched for in Medline, Embase, WanFang Data, medRxiv, bioRxiv, arXiv, SSRN, and Research Square, using SARS-CoV-2, COVID-19, and vaccine as the MeSH terms. Studies with estimates of safety, immunogenicity, and effectiveness from receiving the BIBB-CorV or CoronaVac vaccine that met the predefined screening criteria underwent a full-text review. The Joanna Briggs Institute’s Critical Appraisal Checklist and the Cochrane risk of bias were used for assessment of the quality. A random-effects meta-regression model was applied to identify the potential impact factors on the vaccines’ effectiveness. **Results:** In total, 32578 articles were identified, of these, 770 studies underwent a full-text review. Eventually, 213 studies were included. The pooled occurrence of solicited and unsolicited adverse events after any dose of either vaccine varied between 10% and 40%. The top five commonly reported rare adverse events were immunization stress-related responses (211 cases, 50.0%), cutaneous responses (43 cases, 10.2%), acute neurological syndrome (39 cases, 9.2%), anaphylaxis (17 cases, 4.0%), and acute stroke (16 cases, 3.8%). The majority (83.3%) recovered or were relieved within several days. The peak neutralization titers against the ancestral strain was found within 1 month after the completion of the primary series of either vaccine, with a GMT (geometric mean titer) of 43.7 (95% CI: 23.2–82.4), followed by a dramatic decrease within 3 months. At Month 12, the GMT was 4.1 (95% CI: 3.8–4.4). Homologous boosting could restore humoral immunity, while heterologous boosting elicited around sixfold higher neutralization titers in comparison with homologous boosting. The effectiveness of receiving either vaccine against death and severe disease was around 85% for both shortly after the primary series. At Month 12, the protection against death did not decline, while the protection against severe disease decreased to ~75%. **Conclusions:** Both the BIBP-CorV and CoronaVac inactivated vaccines are safe. Sustained vaccine effectiveness against death was determined 12 months after the primary series, although protection against severe disease decreased slightly over time. A booster dose could strengthen the waning effectiveness; however, the duration of the incremental effectiveness and the additional benefit provided by a heterologous booster need to be studied.

## 1. Introduction

The severe acute respiratory syndrome coronavirus 2 (SARS-CoV-2 virus) has been raging around the world for nearly 3 years since it was characterized as a pandemic by the World Health Organization (WHO) in early 2020. Vaccines have been developed in great hope to help humans survive this disaster. As of November 2022, in addition to 10 COVID-19 vaccines approved for emergency use by the WHO worldwide, 172 and 199 vaccine candidates are being developed with different technologies and are in the clinical and preclinical stages, respectively [1]. However, in spite of high vaccine coverage and promising vaccine effectiveness, almost all countries and regions have experienced a large surge in COVID-19 cases in 2022 [2].

To date, eight COVID-19 vaccines have been approved for emergency use in China, including five inactivated whole-virion vaccines, two subunit protein vaccines, and one adenovirus Type 5 vector vaccine. Of these, three Chinese-made COVID-19 vaccines have been added to the WHO’s Emergency Use Listing (EUL), including two inactivated vaccines [3,4] (BIBP-CorV and CoronaVac) and an adenovirus Type 5 vector vaccine [5] (Ad5-nCoV-S). These three all participated in the COVAX Facility and contributed to the goal of more equitable vaccine distribution [6]. Their easy storage requirements make them highly suitable for low-resource settings due to the characteristics of inactivated vaccines. As of 3 November 2022, 3.44 billion doses of vaccines were rolled out over China, resulting in the coverage of the primary series and booster jabs being 90% and 70%, respectively, in the target age groups [7]. Of these, inactivated vaccines, especially the BIBB-CorV and CoronaVac vaccines, accounted for 95% of doses. Moreover, both vaccines contributed almost half of the COVID-19 vaccine doses delivered globally and have been enormously important in fighting the pandemic [8,9,10,11].

Studies conducted worldwide have demonstrated the good safety profiles and the promising efficacy of the BIBP-CorV and CoronaVac vaccines against the ancestral Wuhan strain in the short-term [12,13,14]. However, after demonstration of the efficacy of the BIBP-CorV and CoronaVac vaccines, the world experienced several epidemic waves caused by the emergence of new globally dominant variants, namely the alpha, delta, and omicron strains. Thus, syntheses of knowledge based on studies that focused on the performance of inactivated vaccines in the real world [15], such as the occurrence of rare adverse events, protection against different variants, protection in elderly, the dynamics of humoral immunity, and effectiveness, are extremely important to inform vaccine policies in a timely manner. Therefore, we carried out a living systematic review that allowed for the constant updating of new emerging evidence and refinement of the methodological quality as studies’ results are reported [16], focusing only on the two Chinese-made inactivated COVID-19 vaccines.

## 2. Materials and Methods

This systematic review was registered at the Inplasy registration website (registration number: INPLASY202470021) in accordance with the PRISMA guidelines.

### 2.1. Search Strategy and Selection Criteria

Studies on COVID-19 vaccines based on human subjects published between 1 May 2020 and 31 May 2022, were identified following the PRISMA guidelines for systematic reviews [17]. Scientific articles published in English and Chinese languages were sought for through searching Medline, Embase (which contains preprints in medRxiv and bioRxiv), and WanFang Data (a Chinese literature service platform). Standardized medical subject heading (MeSH) terms, namely SARS-CoV-2, COVID-19, and vaccine, were established for searching. Considering the rapid mutation of the virus and the resulting changes in the vaccines’ effectiveness in the population, to ensure timely dissemination of the characteristics of the Chinese-made vaccines, preprint studies were searched for on the arXiv servers using the search term “((SARS-CoV-2) OR (COVID-19)) AND (vaccine)”. In addition, SSRN (https://www.ssrn.com/ (accessed on 31 May 2022)) and Research Square (https://www.researchsquare.com/ (accessed on 31 May 2022)) were searched for preprint literature containing the words “((SARS-CoV-2) OR (COVID-19)) AND (vaccine)” in the title, abstract, and keywords, which were also downloaded. The search was initiated on 2 June 2022, with no language restrictions. Prior to the literature search, a pilot study was conducted to refine the MeSH terms and combinations thereof, especially when the terms were translated to Chinese, prior to searching the non-English electronic databases.

Exclusion criteria included studies not related to COVID-19 vaccines; COVID-19 vaccine-related non-clinical studies; COVID-19 vaccine-related clinical studies but not including Chinese vaccines; COVID-19 vaccine-related clinical studies that did not provide numerators and denominators; those with a sample size of <100 participants per arm or <50 participants per arm for effectiveness studies and immunogenicity studies, respectively; immunogenicity studies that did not test neutralizing antibodies, either for the live virus or a pseudovirus; and effectiveness studies with only a clinical definition of syndromic cases. In addition, since this review focused on real world data, the prelicensure clinical trials were excluded.

### 2.2. Review Strategy

Endnote^®^ (version 20, Thomson, Inc., Philadelphia, PA, USA) bibliographic software was used to create an electronic library of the citations identified in the database searches, and duplicate records were deleted. All remaining duplications were eliminated manually during the following screening. Each study was assigned a unique identification code to enable tracking of the reviews and analyses after screening of the title/abstract. Trained reviewers from the teams of Fudan University and China Centre for Disease Control and Prevention (China CDC) performed the screening of the title/abstract, full-text screening, appraisal of the quality, and subsequent data extraction independently. All discrepancies were resolved by consensus between Fudan University’s and China CDC’s teams.

Appraisals of the quality were carried out according to the study design. To be specific, the Joanna Briggs Institute (JBI) Critical Appraisal Checklist for case reports, case serials, and cross-sectional studies was used accordingly [18]. The tools for detecting the risk of bias developed by the Cochrane Bias Methods Group and the Cochrane Non-Randomised Studies Methods Group were applied for assessing the quality of case–control and cohort studies (ROBINS-I, risk of bias in non-randomized studies of interventions) [19] and clinical trials (RoB 2, a revised tool for assessing risk of bias in randomized trials) [20]. The overall risk of bias for each domain was rated as low, moderate, and high with regard to the algorithms of each assessment tool. In detail, for the JBI Checklist, the studies were classified as follows: high methodological quality (>5 ‘‘yes’’ responses), moderate methodological quality (3–4 ‘‘yes’’ responses), or low methodological quality (0–2 ‘‘yes’’ responses). For ROBINS-I and RoB 2, high, moderate, and low methodological quality corresponded to a low, moderate, and high risk of bias.

### 2.3. Data Extraction and Analytical Strategy

Data were extracted from four dimensions, including general information, safety, neutralizing antibodies, and effectiveness. General information, including the study design, study period, study site, study population, the vaccine used, and vaccination regimens (primary, homologous booster, and heterologous booster) were collected from each article that fulfilled the selection criteria, regardless of the study’s objectives. The age was aggregated into children/adolescents (<16 or 18 years when available), adults (<60 years), and the elderly (≥60 or 65 years when available). For the primary series of immunization, of the Chinese-produced vaccines, the BIBP-CorV (produced by Sinopharm) and CoronaVac (produced by Sinovac) inactivated vaccines were predominant for both domestic and overseas use. Therefore, this meta-analysis of the primary series was performed exclusively on the basis of the two inactivated vaccines. In contrast, for the analysis of booster doses, both homologous and heterologous booster doses were included. A homologous booster was defined as an additional dose after the primary series with two doses of the BIBP-CorV or CoronaVac inactivated vaccines; and a heterologous booster was defined as an additional dose of a subunit vaccine, adenovirus vector vaccine, or mRNA vaccine following a primary two-dose series of the BIBP-CorV or CoronaVac inactivated vaccines. In terms of the vaccines’ performance, notable differences between the BIBP-CorV and CoronaVac inactivated vaccines were not observed [21,22]. Moreover, publications on the immunogenicity and effectiveness were few. We therefore summarized our analyses according to the receipt of either of the BIBP-CorV or CoronaVac inactivated vaccines rather than analyzing the performance of the two vaccines separately. Similarly, because of the limited number of eligible studies on immune responses to vaccination, we considered serum neutralizing antibody responses only to the ancestral strain, the delta strain, and the omicron strain.

For assessment of the vaccines’ safety, the incidence of solicited and unsolicited adverse events was directly abstracted as numerators and denominators. In addition, rare adverse events (defined as an unsolicited event with an occurrence of between one episode per 100,000 doses and one episode per one million doses [23]) reported exclusively from case reports and case serials were also collected. With regard to the evaluation of neutralizing antibody responses, the interval from the last dose of the primary vaccination or booster, as appropriate; the strain in the assay challenge (both for live SARS-CoV-2 and pseudovirus) for the neutralizing test; the seropositive rate; and the geometric mean titer of neutralizing antibodies were recorded. The neutralizing antibody response was analyzed by age group (all age groups, adults, elderly, and children), the challenging strain (ancestral, delta, and omicron), the vaccination regimen (primary course, homologous boosters, and heterologous boosters). Considering the waning immunity over time after the vaccine dose, the antibody titers were pooled at different time points, either for the primary series or booster injection, when the data were available and sufficient. Since this meta-analysis aimed to define the magnitude and decay of immunity elicited by the vaccine alone, studies in which immune responses might have been contaminated by responses to natural infection were excluded from the synthesis of the data.

For analyses of the vaccines’ effectiveness, the reported odds ratio, risk ratio, incidence rate ratio or hazard ratio, and 95% CI at each time interval after the full primary series were extracted. We focused on protection by the inactivated COVID-19 vaccines against severe disease and death, rather than protection against asymptomatic infection, and mild/moderate disease. Since some studies merged severe disease with hospitalization, which could not be disaggregated, we considered severe disease with or without hospitalization together in this analysis. Potential factors that might affect the estimates of the vaccines’ effectiveness were also extracted and accounted for in the synthesis of the vaccines’ effectiveness, including previous natural infections in the study population, the follow-up period, age, the predominant strains during the study period, the vaccination regimens, and the interval between the first and second doses in the primary series. To calculate the follow-up period after primary or booster vaccination, we accepted the upper boundary of either the interquartile range (IQR) or of the 95% confidence interval (CI) as the time interval since the final dose of the primary series or boosting injection; when these statistics were not provided, the time interval since the final dose of the primary series or boosting injection was calculated roughly by subtracting the start date of vaccination from the end date of the study in the study population. Because a remarkable difference in the neutralizing antibody titers elicited at 0–14 days and 0–21 days, the schedule of the primary series was observed [24]; the intervals between the first and second doses in the primary series were thus extracted and introduced into the corresponding analysis.

### 2.4. Statistical Analysis

Considering the inter-study variance, the I^2^ statistic was used to present the heterogeneity among studies included in the final analysis. If I^2^ ≥ 50%, high heterogenicity was indicated, and thus random-effects models were used to calculate the point estimate and 95% confidence interval for the summary measures; otherwise, a fixed-effects model was used [25]. Pooled proportions were computed with the inverse variance method using the variance-stabilizing Freeman–Tukey double arcsine transformation [26]. Confidence intervals (CIs) for individual studies were calculated using the Wilson score CI method with continuity correction [27]. For pooling of the means of continuous variables (ORs and GMTs) and their 95% CIs, a logarithmic transformation was applied. Summary-level meta-regression [28,29] for the vaccines’ effectiveness was performed using the random-effects model with the maximum likelihood estimation method to identify factors impacting the outcome from the following variables included in the model: vaccination status (primary series, booster vaccination), interval since last dose, prior infection, proportion of elderly, and interval between the two primary injections. The outcome variable was expressed as log of 1 minus the vaccine’s effectiveness. The publication bias of the studies was assessed using funnel plots, where an asymmetrical distribution of studies was suggestive of bias [30]. Sensitivity analyses were carried out to investigate the influence of the studies’ quality on the pooled effectiveness of the vaccines after the full primary series by removing studies with low quality.

All data were double-entered into custom-made data entry programs based on an electronic data capture system (RIEHEN Solutions Inc., Xi’an, China). The data management program included checks of the range and consistency. All statistical analyses were performed using R software version 4.2.1 (R Foundation for Statistical Computing, 2022), with the package meta [31], and SAS program version 9.4 (SAS Institute Inc., Cary, NC, USA) in appropriate. A *p*-value < 0.05 (2-tailed) was considered to be statistically significant.

## 3. Ethics

This study was reviewed and approved by the Institutes of Biomedical Sciences Institutional Review Board, Fudan University.

## 4. Results

### 4.1. Selection and Characteristics of the Studies

**Figure 1:**
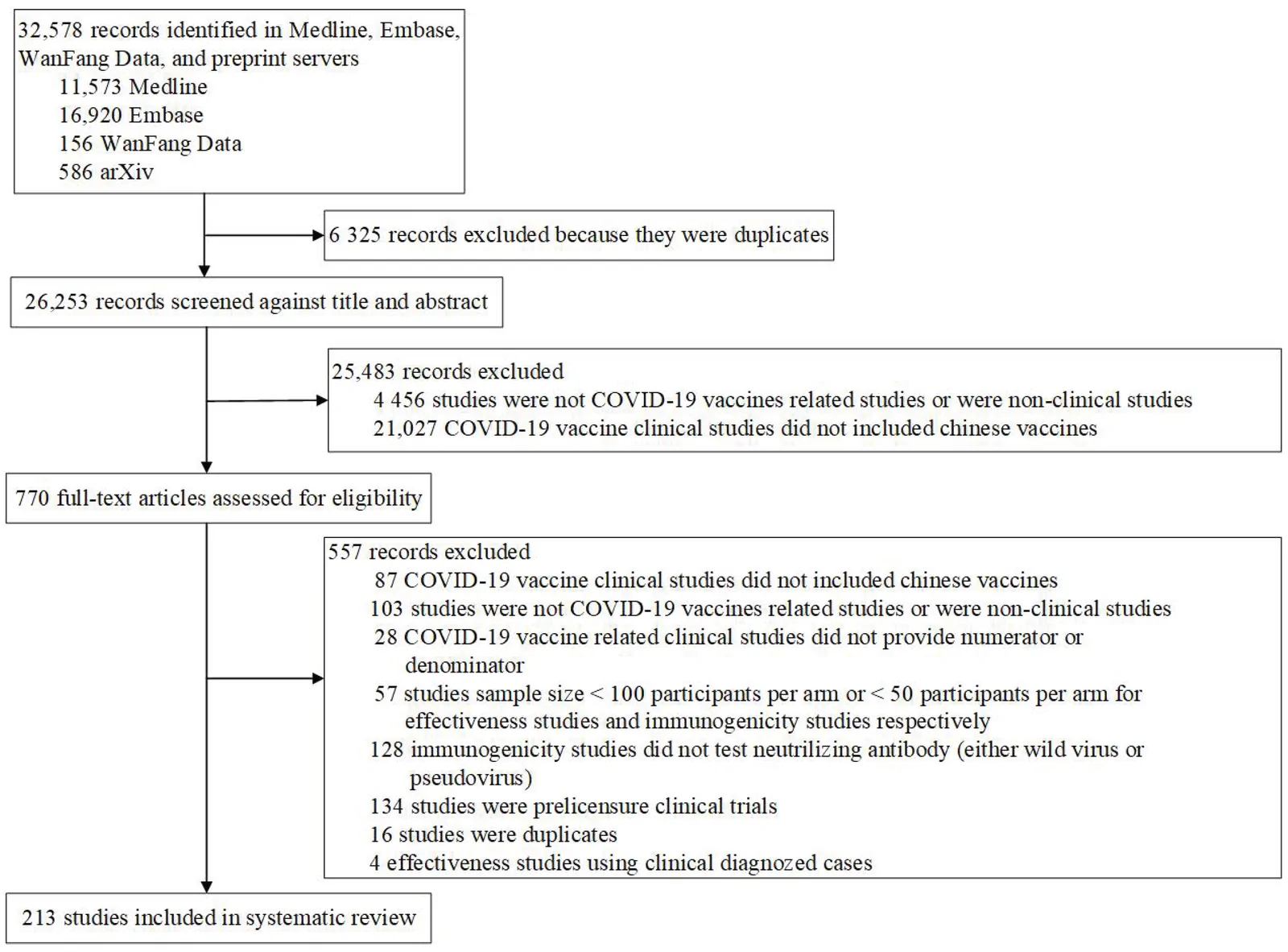
Selection of studies for inclusion in the review.

In total, 32,578 articles published or placed online were identified after systematically searching multiple data sources, including preprint servers. After removing duplicates, 26,253 articles were evaluated by screening the title/abstract and full text, and, eventually, 213 articles were included in final analysis (Figure 1). Of these, 191 (89.6%) and 22 (10.4%) articles came from peer-reviewed databases and preprint servers, respectively. The number of articles providing information on the safety, immunogenicity, and effectiveness were 170 (79.8%), 21 (9.7%), and 33 (15.5%), respectively. Out of these eligible articles, data generated from China were found in 44 (25.9%, 44/170), 17 (81.0%, 17/21), and 6 (18.2, 6/33) articles, respectively. The majority used analytical observation designs (55.8%), followed by case report designs (33.3%). Most data were generated from WHO’s Western Pacific, Europe, and Americas regions, and no articles were reported from Africa. Articles with high and medium quality accounted for 83.1% (Table 1). The number of studies presenting data on heterologous boosters included two (Cansino Ad5-nCoV-S vaccine and the Zhifei longcom recombinant vaccine), seven (ChAdOx1-S recombinant vaccine, the BioNTech BNT162b2 vaccine, the Moderna mRNA-1273 vaccine, the Zhifei longcom recombinant vaccine, and the Cansino Ad5-nCoV-S vaccine), and three (ChAdOx1-S recombinant vaccine, and BioNTech BNT162b2 vaccine) assessing safety, immunogenicity, and effectiveness, respectively. High heterogeneity was observed among the studies included, and the I^2^ varied considerably for assessments of safety (18.3–99.1%), immunogenicity (44.0–99.6%), and the vaccines’ effectiveness (34.3–93.0%). Publication bias was examined by using the funnel plot coupled with Egger’s test, and statistical significance was deduced from studies on effectiveness against severe disease/hospitalization and death (*p* < 0.05) (Supplementary Figure S1a,b). In light of the heterogeneity across these eligible articles, the random-effect model was applied throughout the syntheses.

### 4.2. Safety

**Figure 2:**
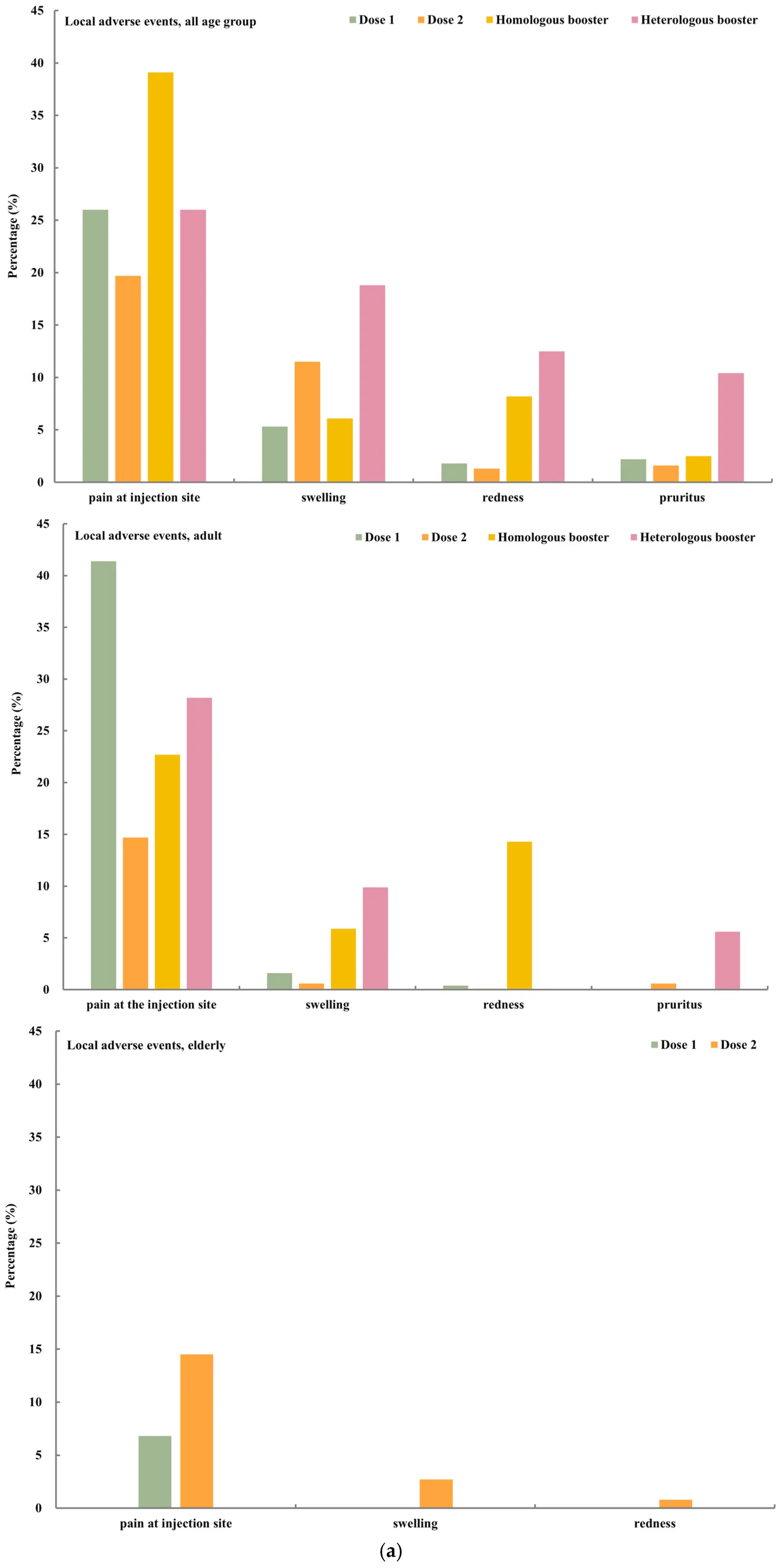

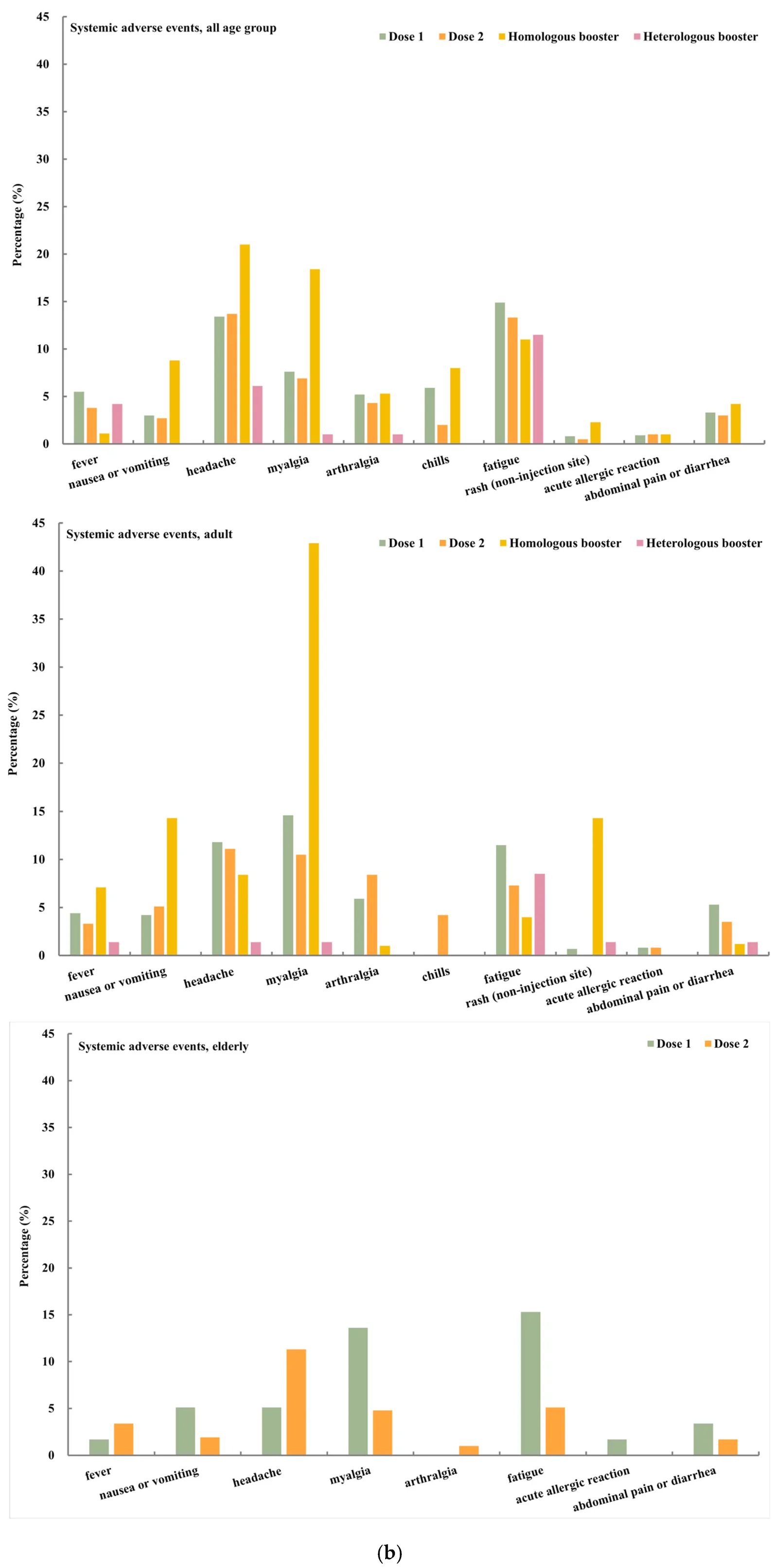

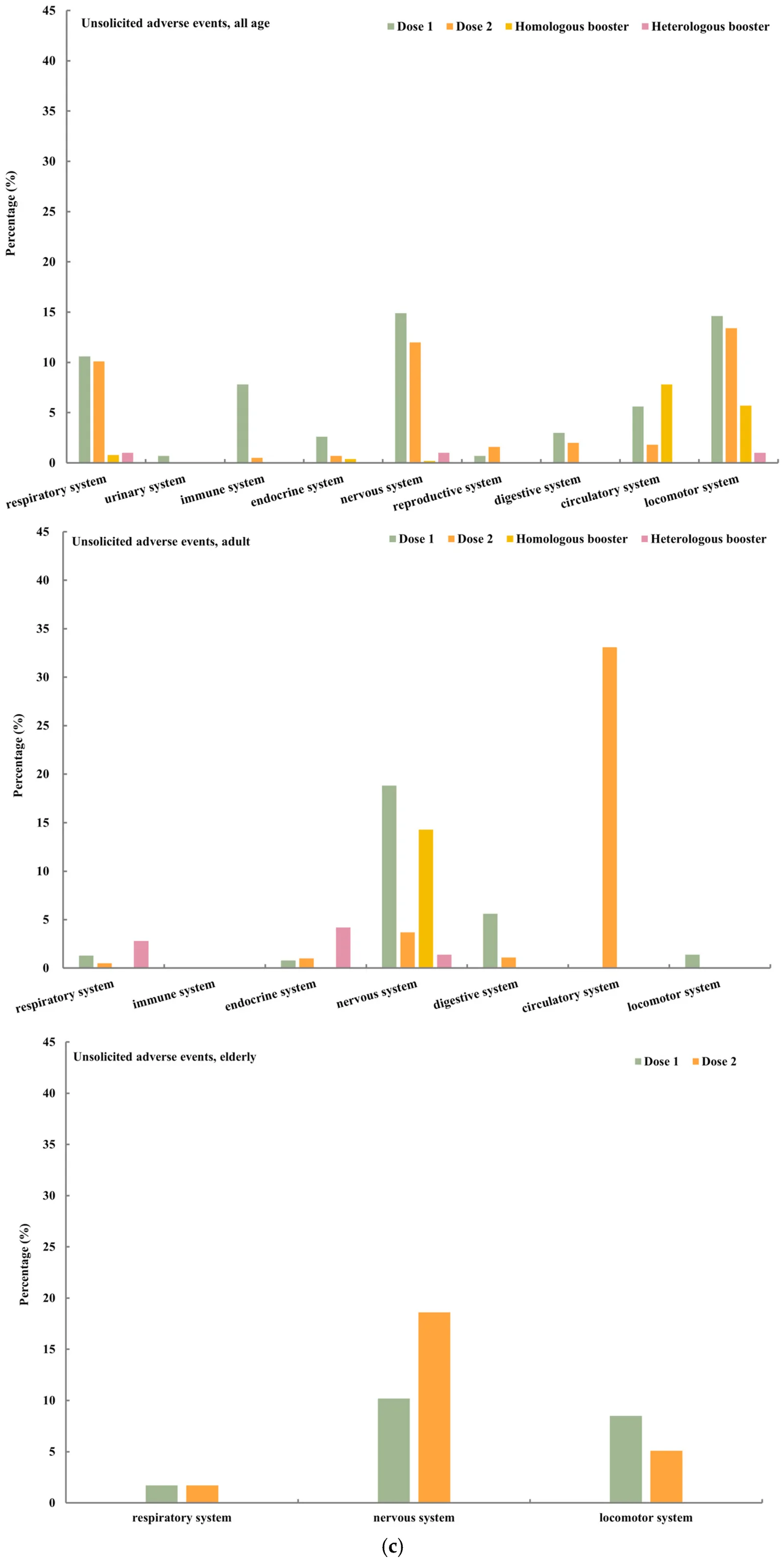
Adverse events after vaccination among three populations (all age, adults, elderly). (**a**) Solicited local adverse events. (**b**) Solicited systematic adverse events. (**c**) Unsolicited adverse events.

Among the 171 articles providing data on the vaccines’ reactogenicity for the solicited events, the most commonly reported local adverse event was pain at the injection site (39.1%; 95% CI: 22.6–55.7%), followed by swelling (6.1%; 95% CI: 2.4–9.8%) and redness (8.2%; 95% CI: 3.2–13.1%), while the most commonly reported systemic reactions were headache (21.0%; 95% CI: 1.1–40.9%), followed by fatigue (11.0%; 95% CI: 1.5–20.4%), myalgia (18.4%; 95% CI: 4.0–32.8%), arthralgia (5.3%; 95% CI: 0–10.7%), and fever (1.1%; 95% CI: 0.4–1.8%). Overall, significant differences in the pooled rates between the first dose and the second dose were not observed, whereas a higher occurrence after homologous booster doses was noticed in comparison with the primary series (Figure 2a,b). There were few studies (three articles, 1.8%) focused on the safety of the elderly. In general, remarkably lower incidence rates of both local and systemic adverse events were observed in the elderly compared with that of all age group (Figure 2a,b). For unsolicited adverse events, the most frequently reported events were in the nervous system (12.0%; 95% CI: 5.6–18.4%), the locomotor system (13.4%; 95% CI: 4.2–22.5%), and the respiratory system (10.1%; 95% CI: 5.4–14.7%). In contrast to solicited adverse events, higher rates of unsolicited adverse events were reported after the primary series, rather than after booster doses (Figure 2c). Again, the occurrence of unsolicited events was lower for symptoms related to the nervous system (18.6%; 95% CI: 13.7–23.6%), the locomotor system (5.1%; 95% CI: 2.3–7.9%), and the respiratory system (1.7%; 95% CI: 0–3.3%) in the elderly (Figure 2c).

There were 89 articles using case reports or case series designs, reporting 422 episodes of rare adverse events, with an average age of 48.1 years (95% CI: 45.5–50.8 years). More adverse events were reported in males (60.0%), and more were reported after the first dose (70.5%), followed by after the second dose (26.7%), and very few were reported after the third dose (2.8%). The average interval for onset after vaccination was 7.4 days (95% CI: 5.9–8.9 days). The top five commonly reported rare adverse events were immunization stress-related responses (211 cases, 50.0%), cutaneous responses (43 cases, 10.2%), acute neurological syndrome (39 cases, 9.2%), anaphylaxis (17 cases, 4.0%), and acute stroke (16 cases, 3.8%) (Supplementary Table S1). In those that reported cutaneous responses, most (22 cases, 51.2%) were pityriasis rosea, while episodes of acute neurological syndrome were mainly diagnosed as Bell’s palsy (28 cases, 71.8%). Out of 422 episodes, the majority (83.3%) recovered or improved, whereas 6.2% and 1.1% failed to recover or died, respectively. For 9.5%, the outcome was not clear.

There was only one article reporting the safety profile after the primary series in adolescents. Pain at the injection site occurred in 54.5% (95% CI: 45.7–63.3%) and 52.8% (95% CI: 44.0–61.7%) of vaccinees after the first and second doses, respectively [32]. For the safety profile in pregnant women, only one study was eligible and included [33]. According to the data from SI-EAPV dataset of the Brazilian Ministry of Health [34], among pregnant women who received at least one dose of a COVID-19 vaccine, the incidence rate of maternal, systemic, and local adverse events was 10.3 episodes/100,000 doses, 50.3 episodes/100,000 doses, and 4.4 episodes/100,000 doses, respectively, in those who received the CoronaVac vaccine compared with 9.7 episodes/100,000 doses, 89.4 episodes/100,000 doses, and 15.5 episodes/100,000 doses in those who received the Pfizer mRNA vaccine. The most common maternal adverse events were spontaneous abortion (4.8% for CoronaVac vs. 4.9% for Pfizer), pregnancy bleeding (3.2% for CoronaVac vs. 0.5% for Pfizer), neonatal death (1.6% for CoronaVac vs. 1.6% for Pfizer), and premature birth (0.5% for CoronaVac vs. 0.5% for Pfizer).

### 4.3. Immunogenicity

**Figure 3:**
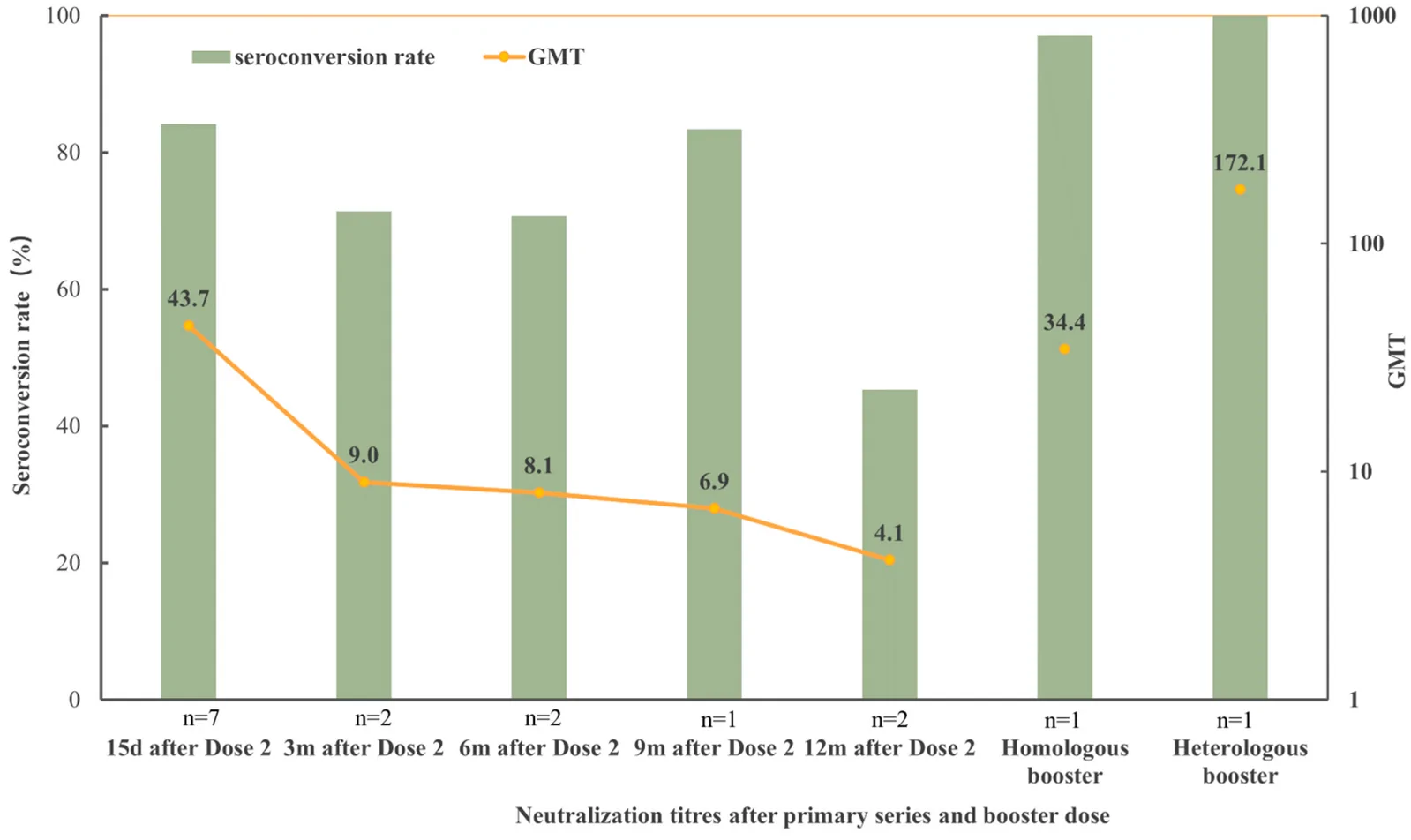
Neutralizing antibodies for the ancestral strain of the live SARS-CoV-2 virus after the primary series and boosters. The bar graphs indicate the trend of the seroconversion rate after two doses of inactivated vaccinations or booster immunization. The line graph connected by yellow dots indicates the trend of GMT after receiving two doses of the inactivated vaccine; the dots for booster immunization are not connected, just displayed.

Of 21 eligible studies, more studies (33.3%) measured the live virus neutralizing antibody in adults. Only one study provided the neutralizing antibody titers against the alpha strain in the elderly with a pseudovirus neutralization assay, and thus was not included in the aggregation. Some studies contributed more than one timepoint, and some reported serum neutralizing antibody titers against variants after vaccination. Overall, 2–4 weeks after the primary series, the pooled seroconversion rate was 84.2% (95% CI: 76.1–92.3%), with a GMT of 43.7 (95% CI: 23.2–82.4) against the ancestral strain measured by a live virus neutralization assay for these adults, and it decreased to 71.4% (95% CI: 21.6–100%), 70.7% (95% CI: 36.1–100%), 83.4% (95% CI: 79.7–87.1%), and 45.3% (95% CI: 19.8–70.7%) at Months 3, 6, 9, and 12, respectively, after completion of the primary series. Accordingly, neutralizing antibody titers waned over time, with a dramatic decrease occurring in the first 3 months after the completion of the primary series (Figure 3). Similar results were also observed with neutralizing antibody measured by pseudovirus assays.

In addition to the immunogenicity against the ancestral strain, a few studies reported the neutralizing antibodies against the delta and omicron strains. Regarding the immunogenicity of the primary series, at 14 to 30 days after the completion, compared with the ancestral strain, a lower antibody response measured by neutralization of the live virus was detected against the delta strain (seroconversion rate: 84.2% vs. 41.7%; GMT: 43.7 vs. 21.2), while in terms of neutralization of the pseudovirus, the seroconversion rate against the ancestral, delta, and omicron strains was 89.8% (95% CI: 84.8–95.2%), 23.4% (95% CI: 13.9–32.8%), and 18.2% (95% CI: 3.2–33.2%), with a GMT of 21.2 (95% CI: 16.2–27.6), 19.6 (95% CI did not provide), and 8.5 (95% CI: 2.4–29.9), respectively. Regarding the antibody response elicited by a booster dose, 14 days after the injection, 20-fold (1409.5, 95% CI: 905.4–2194.5 vs. 69.6, 95% CI: 44.8–108.1) and 13-fold (330.2, 95% CI: 212.1–514.1 vs. 24.6, 95% CI: 18.1–33.5) higher neutralizing antibody titers against the delta and omicron strains were measured for heterologous boosters, in comparison with homologous boosters (Supplementary Table S2).

### 4.4. Effectiveness

**Figure 4:**
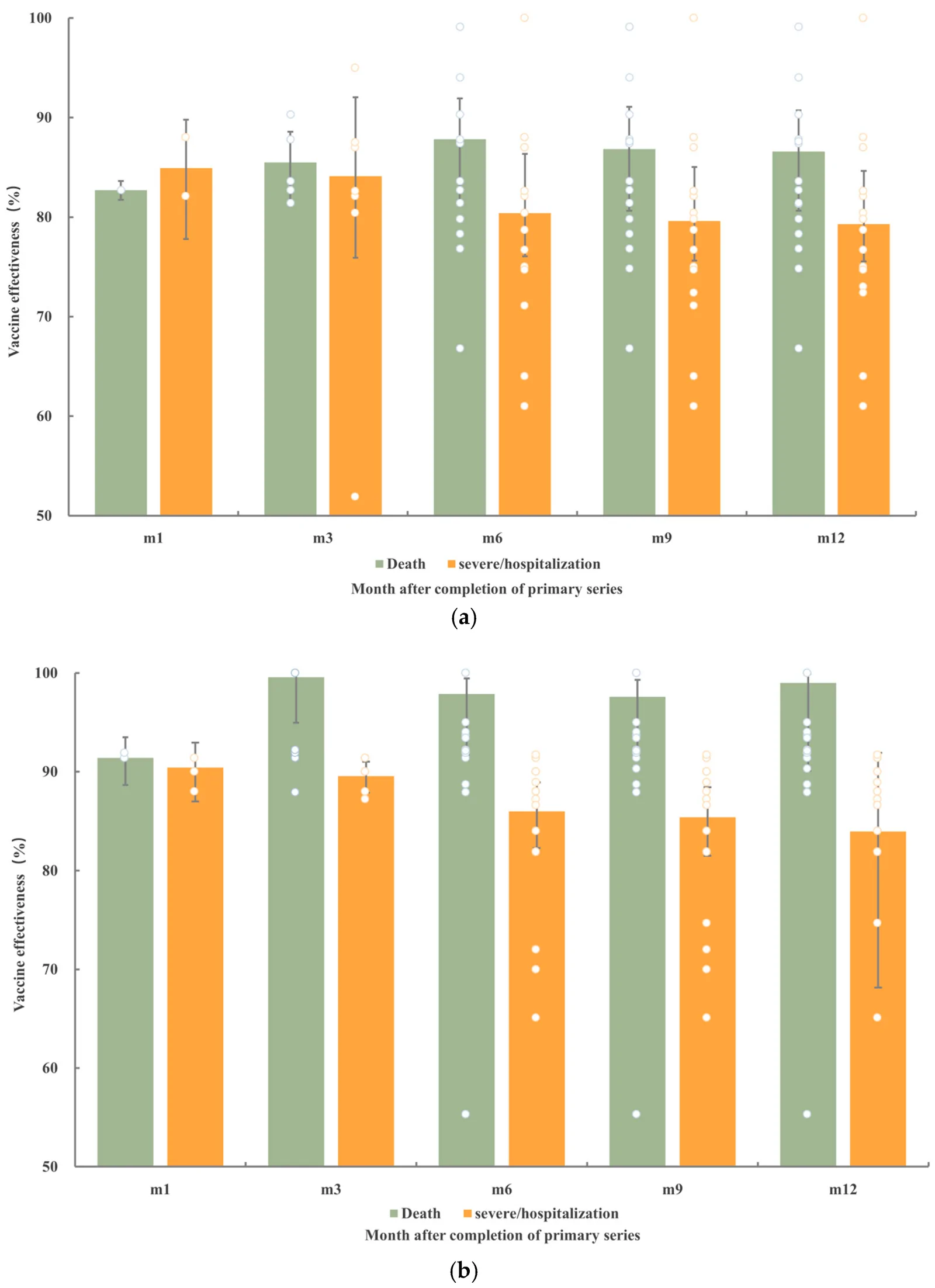
Duration of the vaccine’s effectiveness conferred by the primary series. (**a**) People ≥16 years of age; (**b**) people ≥16 and <60 years of age. The dots in the bar chart show the effectiveness of the vaccine against death and severe infection/hospitalization in the studies in the real word we collected at different periods after completion of the primary series.

Thirty-three studies contributed to the investigation of the vaccines’ effectiveness against laboratory-confirmed COVID-19 infections. Of these, 12 (36.4%) studies focused on infection, including asymptomatic and symptomatic infection, rather than severe disease/hospitalization and death, and thus they were not included in this analysis. In the remaining 21 studies, 9, 13, and 3 studies investigated the effectiveness of COVID-19 vaccines in the adults, elderly, and children/adolescents, respectively. Compared with adults who did not receive any dose of a COVID-19 vaccine, those who completed the primary series exhibited 83.9% protection (95% CI: 68.1–91.9%) against severe disease/hospitalization caused by the ancestral, gamma, delta, or omicron strain, and 99.0% protection (95% CI: 90.1–99.9%) against death caused by the ancestral, delta or omicron strain. Somewhat lower protection conferred by the primary series was found in the elderly (Figure 4a,b). In a comparison of the elderly who did not receive any dose of a vaccine and those who completed the primary series, the vaccines’ effectiveness against severe disease/hospitalization and death caused by the ancestral, delta, and omicron strains was 74.8% (95% CI: 65.5–81.6%) and 88.3% (95% CI: 77.7–93.9%), respectively. Compared with primary vaccination, one dose of a homologous booster resulted in a reduction of 81.8% (95% CI: 40.6–94.4%) and 91.0% (95% CI: 78.0–96.3%) in the occurrence of severe disease/hospitalization caused by the omicron strain in adults and the elderly, respectively. In children and adolescents, a reduction in the outcomes of COVID-19 provided by the primary series against severe illness, critical illness, and death was 86.0% (95% CI: 66.3–94.2%), 93.8% (95% CI: 85.7–97.3%), and 64.0% (95% CI: 0–88.3%), respectively. The protection waned over time, regardless of the clinical outcome of infection and the age group (Figure 4a,b). For a sensitivity analysis, after removing studies with low quality, the effectiveness of the vaccine conferred by the primary series against severe disease/hospitalization and death was 88.1% (95% CI: 79.5–93.1%) and 99.2% (95% CI: 95.5–99.8%), respectively, in adults, and 73.8% (95% CI: 61.5–82.2%) and 88.6% (95% CI: 75.0–94.8%) in the elderly, respectively.

Meta-regression was performed to identify the potential factors affecting the vaccines’ effectiveness against death, severe infection, and/or hospitalization (Table 2). In total, 19 studies with 37 estimates of the vaccines’ effectiveness were included. Better vaccine protection was predicted by a booster injection after a primary series (*p* < 0.05), while a significant association was not detected between the vaccines’ effectiveness and the interval since the last dose, the interval between the two primary injections, prior infection, the proportion of elderly, and the different variants of concern.

## 5. Discussion

Soon after the launch of COVID-19 vaccines in early 2021, two issues have plagued policy makers: the duration of the vaccines’ effectiveness and the protection against variants of concern (VOCs) conferred by vaccines based on the ancestral strain. Several studies have reported that the immunity conferred by internationally implemented mRNA and adenovirus vector vaccines waned over time after the primary series [35,36,37,38,39]. In China, a significant decline over time in the neutralization titers elicited by the BIBP-CorV and CoronaVac vaccines was also observed in later 2021 [40,41], while estimates of the duration of the vaccines’ effectiveness were sparse due to the Zero COVID policy.

This systematic review synthesized data on the performance of the BIBP-CorV and CoronaVac vaccines derived from studies from WHO’s five regions, except for the African continent, and generated several main findings. First, within 12 months after the primary series, these two inactivated vaccines were effective and consistently conferred protection against death and severe disease (including hospitalization) during surges of infection by the SARS-CoV-2 ancestral strain and the ensuing variant strains, though a slight decline over time was observed in the protection against severe illness. Interestingly, this decline was consistent with the dramatic decrease in pooled vaccine-induced neutralization titers against the live SARS-CoV-2 virus within 3 months. Generally, the effectiveness against symptomatic infection was associated with neutralization titers elicited by vaccination [42,43]. However, the mechanism protecting against severe infection might be different, and SARS-CoV-2-specific T cell responses are known to play an important role in clearing intracellular viruses accounting for severe pathological changes [44,45,46]. The evidence synthesized from our study is in line with the observations in Hong Kong during surges of omicron there. The CoronaVac-elicited antibodies became negative at 4 months after primary vaccination, while Pfizer mRNA vaccine’s antibodies remained positive for 6 months [47]. Both vaccines were estimated to have high effectiveness against severe disease in adults who completed the primary series, and a third doses of either BNT162b2 or CoronaVac provided additional protection against severe disease [9]. However, the effectiveness of the heterologous BNT162b2 booster against severe disease or death is higher than the homologous CoronaVac booster, epically 4 months after the booster [48]. The trend of the vaccines’ effectiveness is consistent with the antibodies.

Second, waning immunity over time and immune escape caused by new variants are the most significant risk factors weakening the vaccines’ effectiveness, while a booster, either homologous or heterologous, can recover the protection. In this analysis, the multivariate meta-regression analysis detected the significant positive association of the vaccines’ effectiveness with booster injections, rather than with the interval since the last dose or the variant, though the duration of restored immunity triggered by boosting needs to be determined in a subsequent analysis of pooled data. Sustainable protection against severe disease and death over 12 months has been discussed previously. It was interesting that a significant association between protection and the variants was not observed. Immune evasion due to mutations of COVID accounting for the variants has been reported frequently, especially the escape caused by the omicron strain, which was confirmed by cross-neutralization assays [49,50,51,52]. The most likely explanation for our findings on inactivated whole virion vaccines might be the broad spectrum of antibodies and T cell responses elicited by these vaccines. Unlike vaccines based only on the spike protein, inactivated vaccines might elicit more diverse T cell responses recognizing distinct structural proteins, such as membrane and nucleoprotein antigens; these wider specificities of the T cell response might be efficiently compensate for the spike mutations characterizing the omicron strain and thus confer better protection against severe or fatal outcomes after infection [53,54]. Alternatively, these findings may have resulted from the inherent limitations of drawing conclusions from relatively small numbers of studies using different methodologies and of differing levels of quality in diverse populations. Consequently, these factors potentially affecting the vaccines’ effectiveness require confirmation in future research [16].

Overall, the pooled magnitudes of solicited and unsolicited adverse events in the studies were low (10–30%). However, they were comparatively higher than the safety profile reported from the Phase 1/2 clinical trials of the BIBP-CorV and CoronaVac vaccines [24,55]. This observation underscores the importance of real-world data and synthesizing real-world evidence. The discrepancies could be explained by high selectivity of participants in clinical trials or, alternatively, by overestimates due to potential reporting bias, as the majority of the studies evaluating adverse events were survey-based. The most common solicited adverse event after the primary series and boosters of inactivated vaccines was pain at the injection site, followed by headache, fatigue, and myalgia, with the occurrence varying between 10% and 40%. The main unsolicited adverse events reported were those in the nervous system, the locomotor system, and respiratory system (around 10–15%), and were mostly limited to the short period after the injection of the first and second doses. In addition, 422 rare adverse events were noticed and reported; majority (83.3%) recovered or were relieved within several days. Though causality between these rare adverse events and vaccination was not fully established, they provided signals for further evaluation [56]. Moreover, the well-recognized neurological complications associated with many forms of vaccines, namely Guillain–Barré syndrome and acute disseminated encephalomyelitis were only reported in two cases, and only in China. Considering that 2.1 billion doses of the BIBP-CorV and CoronaVac vaccines were used overseas, the current synthesis of safety data suggests that the inactivated vaccines are safe in adults and the elderly.

## 6. Limitations

This living systematic review might have several limitations. First, since limited eligible studies were included in the synthesis of immunogenicity and the vaccines’ effectiveness, the robustness of our conclusions may be limited. However, the sample sizes of almost all individual studies used for pooling the average estimates were more than enough to fulfil the requirements of statistical power. Second, few studies evaluated the vaccines’ effectiveness separately or solely in the elderly, pregnant women, and children/adolescents; thus, protection in these populations needs to be further verified. Third, due to the predefined period covered by this analysis, studies on the effectiveness of homologous versus heterologous regimens were lacking, which is an important omission, since heterologous regimens may be more effective in terms of the duration of protection and the ability to protect against viral mutations causing immune escape. Fourth, generally, evaluating the trend of immunogenicity and the effectiveness of vaccines is legitimate only if the included subjects are measured at all time points. However, since we did not contact the authors and relied on the published studies, and since each individual study had its unique follow-up period and observation time points, this assumption was not always fulfilled among the studies evaluated here. Nevertheless, because this is an interim report of a living systematic review, the findings of the ongoing review should be strengthened over time through consideration of more eligible studies. Over time, this review will encompass the rapidly evolving characteristics of the pandemic due to the rapidly mutating SARS-CoV-2 virus, the population’s evolving natural and vaccine-induced immunity, and the use of new vaccination regimens and vaccine combinations, even new vaccines. This ongoing systematic review will thereby provide timely evidence to inform decision-makers and stakeholders.

## 7. Conclusions

In conclusion, our study showed that the pooled occurrence of solicited adverse events and unsolicited adverse events after mass implementation of the BIBP-CorV and CoronaVac inactivated vaccines were low, either for primary series or for homologous/heterologous boosters. Virus neutralization titers in the serum appeared to decline dramatically in the first 3 months after completion of the primary series but were restored by a booster injection. Compared with a homologous booster, a heterologous booster elicited much higher levels of neutralizing antibodies. Sustained effectiveness of the vaccine against death conferred by inactivated vaccines was observed during the 12 months after completion of the primary series, while protection against severe disease declined slightly over time. A booster dose appeared to restore protection in the face of this waning effectiveness. However, the duration of the incremental effectiveness, as well as the additional benefit provided by heterologous booster, need to be determined in subsequent updates of our living systematic review.

## Figures and Tables

**Table 1:** Characteristics of studies included in the meta-analysis.

Characteristics	Number of Studies (%) (*n* = 213)
**Publication year**	
2021	83 (39.0)
2022	130 (61.0)
**Publication status**	
Peer-reviewed, Chinese	2 (0.9)
Peer-reviewed, English	189 (88.7)
Preprint	22 (10.4)
**Study design**	
Case report	71 (33.3)
Case serials	14 (6.6)
Cross-sectional study	52 (24.4)
Case–control study	15 (7.0)
Cohort study	52 (24.4)
Clinical trials	9 (4.2)
**Study objectives ^a^**	
Safety	170 (79.8)
Neutralization titers	21 (9.7)
Effectiveness	33 (15.5)
**WHO regions ^b^**	
Africa	0 (0)
Americas	40 (18.8)
Eastern Mediterranean	39 (18.4)
Europe	48 (22.5)
Southeast Asia	26 (12.2)
Western Pacific	60 (28.1)
**Quality of study**	
High	121 (56.8)
Medium	56 (26.3)
lLw	36 (16.9)

Notes: ^a^ Since some articles might cover more than one objective, the sum of the percentages can thus exceed 100%. ^b^ All 60 studies in the category of the Western Pacific Region were reported from China.

**Table 2:** Potential factors impacting the vaccines’ effectiveness identified by the meta-regression.

Potential Impact Factor	OR (%, 95% CI)	*p* Value
Death as the endpoint (9 studies, 16 estimates)		
** Interval since the last dose**	1.34 (0.08, 22.89)	0.8402
** Interval between the two primary injections**	9.70 (0.44, 212.51)	0.1491
<3 weeks (Ref.)		
≥3 weeks	0.66 (0.11, 4.09)	0.6586
** Prior infection**	1.05 (0.03, 35.98)	0.9771
** Vaccination regimens**		
Primary vaccination (Ref.)	-	-
Homologous booster vaccination	0.08 (0.01, 0.48)	0.0054
** Proportion of elderly**	2.32 (0.27, 19.89)	0.4412
** Variant of concern**		
Before delta (Ref.)	-	-
Delta contained	1.58 (0.01, 191.25)	0.8522
Omicron	1.35 (0.04, 40.85)	0.8633
Severe disease as the endpoint (12 studies, 21 estimates)		
** Interval since the last dose**	1.44 (0.71, 2.91)	0.3123
** Interval between the two primary injections**		
<3 weeks (Ref.)	NA	
≥3 weeks	NA	
** Prior infection**	NA	NA
** Vaccination regimens**		
Primary vaccination (Ref.)	-	-
Homologous booster vaccination	0.26 (0.07, 0.95)	0.0417
** Proportion of elderly**	1.62 (0.34, 7.73)	0.5437
** Variant of concern**		
Before delta (Ref.)	-	-
Delta contained	0.65 (0.11, 3.62)	0.6186
Omicron	0.53 (0.1, 2.96)	0.4706

## Supplementary Materials

The following supporting information can be downloaded at https://www.mdpi.com/article/10.3390/vaccines12070781/s1. Table S1: Reported rare adverse events (Number of studies = 88); Table S2: Seroconversion rates and GMTs of neutralizing antibody after primary and booster vaccination; Table S3: Characteristics of immunogenicity studies included; Table S4: Characteristics of effectiveness studies included; Figure S1a: Funnel plots to detect publication bias for studies on effectiveness against severe disease/hospitalization; Figure S1b: Funnel plots to detect publication bias for studies on effectiveness against death; Figure S2: Forest plot of vaccine effectiveness against SARS-CoV-2 infection stratified by outcome and age group.
