# Supplementary material for: Safety, Immunogenicity, and Effectiveness of Chinese-Made COVID-19 Vaccines in the Real World: An Interim Report of a Living Systematic Review
Source: Vaccines (Basel). 2024 Jul 16;12(7):781. doi: 10.3390/vaccines12070781 (PMC11281383; doi:10.3390/vaccines12070781)

Supplementary Table S1. Reported rare adverse events (Number of studies =88)

**Reported adverse events**	**No. of Episodes (%)**
Immunization stress-related responses	211 (50%)
Cutaneous	43 (10.2)
Acute neurological syndrome	39 (9.2)
Anaphylaxis	17 (4.0)
Acute Stroke	16 (3.8)
Subacute thyroiditis	13 (3.1)
pemphigus vulgaris (PV)	13 (3.1)
Arthritis	6 (1.4)
Tolosa-Hunt syndrome (THS)	5 (1.2)
Vogt-Koyanagi-Harada Syndrome	4 (0.9)
Uveitis	4 (0.9)
Asculitis	3 (0.7)
Herpes zoster	3 (0.7)
Guillain-Barre syndrome	2 (0.5)
Acute Coronary Syndrome	2 (0.5)
Corneal Graft Rejection	2 (0.5)
Deafness	2 (0.5)
lichenplanopilaris（LPP）	2 (0.5)
Vascular retinal findings	2 (0.5)
acral chilblain-like lesions (CLL)	2 (0.5)
Steven-Johnson syndrome	2 (0.5)
transient heart block	2 (0.5)
Acute disseminated encephalomyelitis (ADEM)	2 (0.5)
Type 1 Kounis Syndrome	1 (0.2)
vitiligo	1 (0.2)
Fulminant type 1 diabetes	1 (0.2)
Peripheral ulcerative keratitis (PUK)	1 (0.2)
Symmetrical drug-related intertriginous and flexural exanthema (SDRIFE)	1 (0.2)
Multiple evanescent white dot syndrome (MEWDS)	1 (0.2)
Multifocal choroiditis	1 (0.2)
Graves' disease	1 (0.2)
Coronary Thrombus	1 (0.2)
Iritis	1 (0.2)
Acute thyroiditis and bilateral optic neuritis	1 (0.2)
Acute idiopathic maculopathy	1 (0.2)
acute asthma exacerbation	1 (0.2)
Miller Fisher syndrome	1 (0.2)
Immune-mediated necrotizing myopathy	1 (0.2)
Vaccine-induced immune thrombotic thrombocytopenia（VITT）	1 (0.2)
Ischemic colitis	1 (0.2)
Eosinophilic panniculitis	1 (0.2)
Hemophagocytic lymphohistiocytosis	1 (0.2)
Neuromyelitis optica (NMO)	1 (0.2)
atrial fibrillation	1 (0.2)
Orbital cellulitis	1 (0.2)
Shoulder injury related vaccine administration	1 (0.2)
Relapse of primary membranous nephropathy	1 (0.2)
Symptomatic dermographism	1 (0.2)

Supplementary Table S2. Seroconversion rates and GMTs of neutralizing antibody after primary and booster vaccination

Month after primary series	Variants	Pooled seroconversion rate (%)			Pooled GMT		
		No. of Studies	No. of participants	Rate (95%CI)	No. of Studies	No. of participants	GMT (95%CI)
Live virus neutralization assay							
Primary vaccination							
0.5 – 1 month	ancestral strain	6	2043	84.2 (76.1, 92.3)	7	2164	43.7 (23.2, 82.4)
3 months	ancestral strain	2	363	71.4 (21.6, 100.0)	2	363	9.0 (4.7, 17.6)
6 months	ancestral strain	2	457	70.7 (36.1, 100.0)	2	457	8.1 (5.3, 12.4)
9 months	ancestral strain	1	385	83.4 (79.7, 87.1)	1	385	6.9 (5.2, 9.0)
12 months	ancestral strain	2	536	45.3 (19.8, 70.7)	2	536	4.1 (3.8, 4.4)
Booster vaccination							
Homologous boost at 3-6 months	ancestral strain	1	102	97.1(93.8, 100.0)	1	102	34.3 (30.3,38.9)
Heterologous boost at 3-6 months	ancestral strain	1	192	100.0(99.0, 100.0)	1	96	172.1 (131.7,224.7)
	delta strain	1	96	95.8(91.8, 99.8)	1	96	55.0 (44.5,68.0)
Pseudovirus neutralizing assay							
Primary vaccination							
0.5 – 1 month	ancestral strain	3	1145	89.8(84.4, 95.2)	2	1052	55.9 (43.6, 71.6)
	delta strain	1	77	23.4(13.9, 32.8)	1	77	19.6[a]
	omicron strain	2	369	18.2(3.2, 33.2)	2	369	8.5 (2.4, 29.9)
6 months	ancestral strain	3	581	37.9(33.9, 41.8)	1	581	40.8 (33.7, 49.4)
	delta strain	3	220	43.6(37.1, 50.2)	1	220	15.9 (12.7, 19.9)
9 months	ancestral strain	3	292	18.2(13.7, 22.6)	1	292	< 4
	omicron strain	3	292	2.7(0.9, 4.6)	1	292	< 4
Booster vaccination							
Homologous boost at 6 months	ancestral strain	1	292	98.3(96.8, 99.8)	1	292	294.9 (253.0, 343.6)
	delta strain	1	77	89.6(82.8, 96.4)	1	77	64.8 (53.4, 78.6)
	omicron strain	2	446	78.0(54.0, 100.0)	2	369	22.2 (9.8, 50.4)

Note: a. 95% CI did not present in the article.

Supplementary Table S3. Characteristics of immunogenicity studies included

Author	Country	Neutralization test	Population	Study design	China-made vaccine contained	Sample size	Study duration	Neutralizing strain
Huang et al	China	Live virus neutralization test	18–59 years HIV (PLWH) and HIV-negative individuals	Cross-Sectional Study	received at least 1-dose Sinovac CoronaVac or Sinopharm	327	April and June 2021	wild type
Cheng et al	China	Pseudovirus neutralization test	Age 20-74 years	Cohort study	3-dose BBIBP-CorV	479	26 January 2021 and 6 December 2021	wild type
Feng et al	China	Live virus neutralization test	Age 18–59 years	Randomized, controlled clinical trial	2-dose BBIBP-CorV (0–28)<br>2-dose BBIBP-CorV (0–21)<br>2-dose BBIBP-CorV(0–14)	269<br>270<br>270	January and May 2021	wild type
Zhang et al	China	Pseudovirus neutralization test	ALL-age	An open and parallel-controlled study	2-dose CoronaVac+ CoronaVac<br>2-dose CoronaVac+ ZF2001<br>2-dose CoronaVac+ Ad5-nCoV	904	Dec-21	wild type/ Omicron
Clemens et al	Brazil	Live virus neutralization test	Age ≥18 years	Randomized, controlled clinical trial	2-dose CoronaVac+ChAdOx1 nCoV-19<br>2-dose CoronaVac+BNT162b2<br>2-dose CoronaVac+Ad26.COV2-S	304<br>340<br>306	Between Aug 16, and Sept 1, 2021	D614G
Wang et al	China	Live virus neutralization test	Age 18-59 years	Cross-sectional survey	2-dose BBIBP-CorV or CoronaVac.	1527	March 2021 to December 2021	wild type
Oliveira et al	Brazil	Live virus neutralization test	ALL-age	Prospective cohort study	2-dose CoronaVac	212	between August/2020 and August/2021	Gamma
Li et al	China	Live virus neutralization test	Age 18-75 years	Randomized phase 4 trial	2-dose CoronaVac+ CoronaVac<br>1-dose CoronaVac+ CoronaVac<br>2-dose CoronaVac+ AD5-based vaccine	302	Between May 25 and 26, 2021	wild type/ Delta
Ai et al	China	Pseudovirus neutralization test	Healthy adults	Prospective cohort study	2-dose inactivated vaccines (CoronaVac or BBIBP-CorV) +ZF2001<br>2- doses inactivated vaccines (CoronaVac or BBIBP-CorV)	122	Unknown	wild type/ Alpha /Beta/ Gamma

Yu et al	China	Pseudovirus neutralization test	Healthcare workers (aged 18-59 years)	Cohort study	3-dose BBIBP-CorV	292	Unknown	wild type/ Omicron
Chen et al	China	Pseudovirus neutralization test	Healthcare workers	Prospective cohort study	3-dose CoronaVac	77	During the period from 8 Nov to 14 Nov 2021.	Delta/ Omicron
Assawakosri et al	Thailand	Live virus neutralization test	Age ≥18 years	Prospective cohort study	2-dose CoronaVac+BBIBP<br>2-dose CoronaVac+AZD1222<br>2-dose CoronaVac+ BNT162B2<br>2-dose CoronaVac+ mRNA-1273	224 individuals	September 2021 and December 2021	Delta/ Omicron
Yu et al	China	Pseudovirus neutralization test	healthcare workers	Observational clinical trial	2-dose BBIBP-CorV	1006	Between January 14, 2021 and March 10, 2021	wild type/ Delta
Chen et al	China	Live virus neutralization test	Age 18-59 years	Randomized control trial	2-dose BBIBP-CorV (0–28)<br>2-dose BBIBP-CorV(0–21)<br>2-dose BBIBP-CorV(0–14)	405	Unknown	wild type
Cai et al	China	Live virus neutralization test	People living with HIV	Cross-sectional study	2-dose BBIBP-CorV	143 PLWHs	Unknown	wild type/ Delta
Acevedo et al	Chile	Pseudovirus neutralization test	Healthcare workers	Cohort study	2-dose CoronaVac	53	Unknown	wildtype/Alpha/Lambda/Gamma
N. Angkasekwinai et al.	Thailand	Live virus neutralization test	Health care workers Age ≥18 years	Prospective cohort study	2-dose ChAdOx1<br>2-dose CoronaVac	360	February to July 2021	wild type/ Alpha /Beta/ Delta
Chen et al	China	Pseudovirus neutralization test	Healthcare workers	Cross-sectional study	2-dose CoronaVac	93	Unknown	wild type/Lota/Beta/Gamma
Zhang et al	China	Live virus neutralization test	Aged 18-76 years	Cohort study	CoronaVac	94<br>95	Unknown	Unknown
Zhao et al	China	Live virus neutralization test	Age 18-59 years	Prospective cohort study	2-dose CoronaVac	145	Unknown	wild type
X. YU et al	China	Pseudovirus neutralization test	Age 18-59 years	Cross-sectional study	3-dose inactivated COVID-19 vaccine	200	On December 10, 2021	wild type/ Delta/

Supplementary Table S4. Characteristics of effectiveness studies included

Author	Country	Population	Study design	China-made vaccine contained	Sample size	Study duration	Epidemic strain
Javier et al	Peru	HCW age ≥18 years	Retrospective cohort study	2-dose BBIBP-CorV	606870	Feb 9 to Jun 30, 2021	Gamma
Hu et al	China	Age ≥18 years	Retrospective cohort study	2-dose BBIBP-CorV<br>2-dose CoronaVac	476	July to August 2021	Delta
Cerqueira-Silva et al	Brazil	ALL-age	Test-negative, case-control study	2-dose CoronaVac	213457	July and September 2020 February and June 2021	Alpha, Beta, Delta
Li et al	China	Age 18–59 years	Test-negative case–control study	2-dose inactivated COVID-19 vaccine	366	May to June, 2021	Delta
Jara et al	Chile	Age ≥16 years	Prospective cohort study	2-dose CoronaVac +CoronaVac<br>2-dose CoronaVac+AZD1222<br>2-dose CoronaVac+ BNT162B2	11174257	Feb 2 to Nov 10, 2021	Gamma, Delta
Ma et al	China	Age ≥18 years	Retrospective cohort study	2-dose BBIBP-CorV<br>2-dose CoronaVac<br>1-dose BBIBP-CorV+1-dose CoronaVac	1058	March through May 2021,	Delta
Kang et al	China	Age ≥18 years	Retrospective cohort	2-dose BBIBP-CorV<br>2-dose CoronaVac	12501	Between May and June 2021	Delta
Sritipsukho et al	Thailand	Age ≥18 years	Test-negative case–control study	2-dose CoronaVac+ChAdOx1 nCoV-19<br>2-dose CoronaVac+BNT162b2<br>2-dose CoronaVac	3353	27 July 2021 to 23 October 2021	Delta
Cerqueira-Silva et al	Brazil	Age ≥18 years	Test-negative, case-control study	2-dose CoronaVac+BNT162b2	7863	From 24 February 2020 to 11 November 2021	Alpha, Beta, Delta
Belayachi et al	Morocco	Age ≥18 years	Test-negative, case-control study	2-dose BBIBP-CorV	25768	Between February 01,2021 and October 01 2021	Delta、Beta、Kappa
AlHosani et al	Abu Dhabi, United Arab Emirates (UAE)	Age > 15 years	Test-negative, case-control study	2-dose BBIBP-CorV	154872	Between September 01, 2020 and May 1, 2021	Alpha, Beta
Mirahmadizadeh et al	Iran	Age ≥18 years	Historical cohort study	2-dose BBIBP-CorV	3628857	Feb 9 to Oct 22, 2021	Delta
Mousa et al	UAE	ALL-age	Bivariate case–control panel	2-dose BBIBP-CorV	3782	August–November2021	Delta

Petrovi´c et al	Vojvodina	Age ≥60 Years	Cohort study	2-dose BBIBP-CorV	139858	January–April 2021	Wuhan, Alpha
Rearte et al	Argentina	Age ≥60 Years	Test-negative, case-control, and retrospective longitudinal study	2-dose BBIBP-CorV	1282928	Jan 31, to Sept 14, 2021,	Alpha, Gamma
Sultan et al	Jordan	Health Care Workers	Cross-sectional study	2-dose BBIBP-CorV	2855	Jan to Sept 30, 2021	Delta
Voko' et al	Hungary	Age ≥16 years	Retrospective, observational study	2-dose BBIBP-CorV	3740066	Between 22 January 2021 and 10 June 2021	Alpha
Nabirova et al	Kazakhstan	Age ≥18 years	Retrospective cohort	2-dose CoronaVac	1312948	February 22 to Sept 1, 2021	Delta
Nisar et al	Pakistan:	Age ≥18 years	Test-negative case-control study	2-dose BBIBP-CorV	3187	July 26 to Dec 4, 2021	Delta
Alencar et al	Brazil	Over 75 years	Retrospective, observational study	2-dose CoronaVac	174006	Jan 17 to May 21, 2021	Gamma
Can et al	Turkey	health care workers	Retrospective cohort study	2-dose CoronaVac	2267	Mar 1, to May 31, 2021	Beta
Cerqueira-Silva	Brazil	Age ≥18 years	Test-negative, case-control study	2-dose CoronaVac	913052	From 24 February 2020 to 11 November 2021	Alpha, Beta, Delta
Jara et al	Chile	Age ≥16 years	Prospective national cohort	2-dose CoronaVac	9645302	February 2 through May 1, 2021,	Alpha, Gamma
Jara et al	Chile	children 3–5 years of age	Prospective cohort	2-dose CoronaVac	490694	6 December 2021 through 26 February 2022	Omicron
R. Marra et al	Brazil	HCWs (aged ≥18 years)	Retrospective cohort study	2-dose CoronaVac	13813	January 1, 2021 and August 3, 2021	Alpha, Gamma
Paixao et al	Brazil	pregnant women	Test-negative case-control study	2-dose CoronaVac	19838	March 15, 2021, to October 03, 2021	Gamma, Delta
Ranzani et al	Brazil	Age ≥70 years	Test negative case-control study	2-dose CoronaVac	43755	17 January to 29 April 2021	Wuhan, Gamma
Rifai et al	Indonesia	Health Care Workers-age 18–59 years	Prospective cohort study	2-dose CoronaVac	155	January to September 2021	Delta
Suryatma et al	Indonesia	Age ≥18 years	Test negative case-control study	2-dose CoronaVac	19688	January 13 to June 30, 2021	Wuhan, Delta
Paixao et al	Brazil	Pregnant	Test-negative design study	2-dose CoronaVac	2033	March 15, 2021, and October 03, 2021,	Gamma, Delta

Jara et al	Chile	Age 6-16 years	Prospective national cohort	2-dose CoronaVac	2000000	Between June 27, 2021, and January 12, 2022	Gamma, Delta, Omicron
Florentino et al	Brazil	Age 6-11 years	Case-control cohort	2-dose CoronaVac	17141	January 21, 2022, up to April 19, 2022	Omicron
AlQahtani et al	Bahrain	Age ≥18 years	Case-control cohort	2-dose CoronaVac	1003960	Feb 1 to July 31, 2021	Delta、Beta、Kappa

Figure S1a. Funnel plots to detect publication bias for studies on effectiveness against severe disease/hospitalization

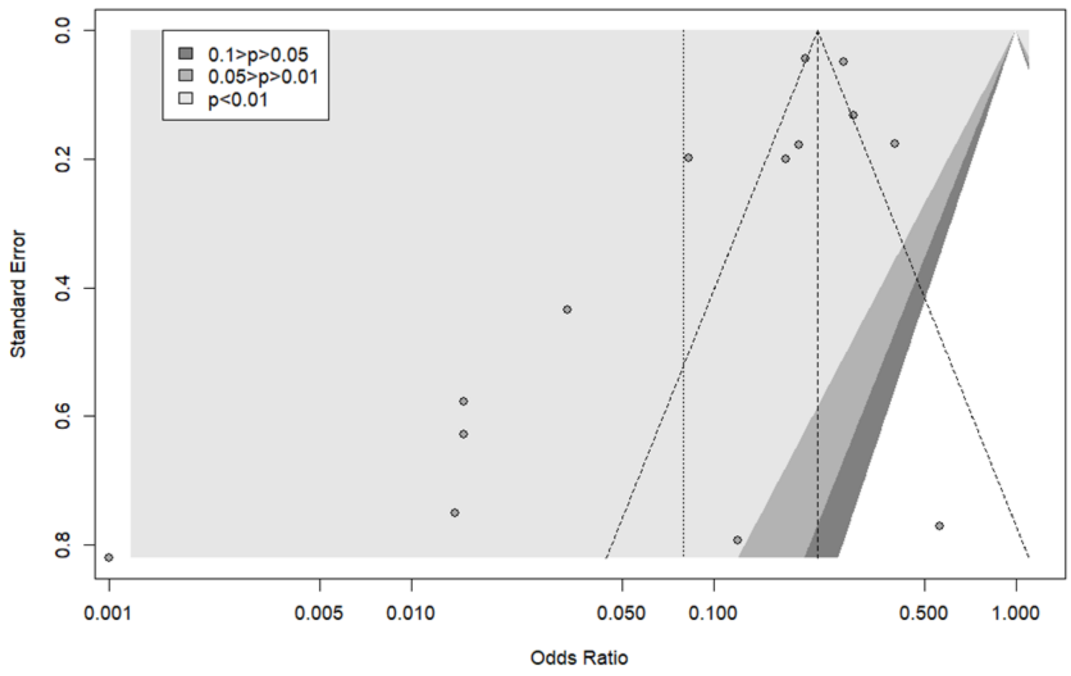

Figure S1b. Funnel plots to detect publication bias for studies on effectiveness against death

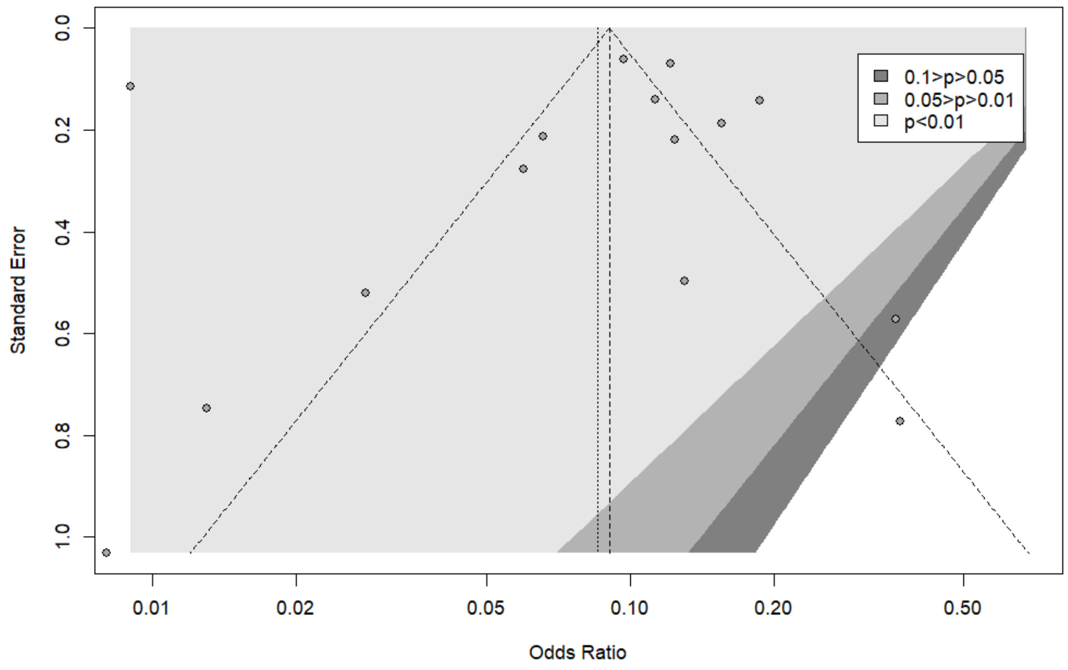

Supplementary Figure S2. Forest plot of vaccine effectiveness against SARS-CoV-2 infection stratified by outcome and age group

Mean VE values represent the mean vaccine protection expressed as percentage. Blue squares and their corresponding lines are the point estimates and 95% confidence intervals (95% CI). Orange diamonds and their corresponding lines represent the pooled VE estimates for each group as well as its 95% CI.

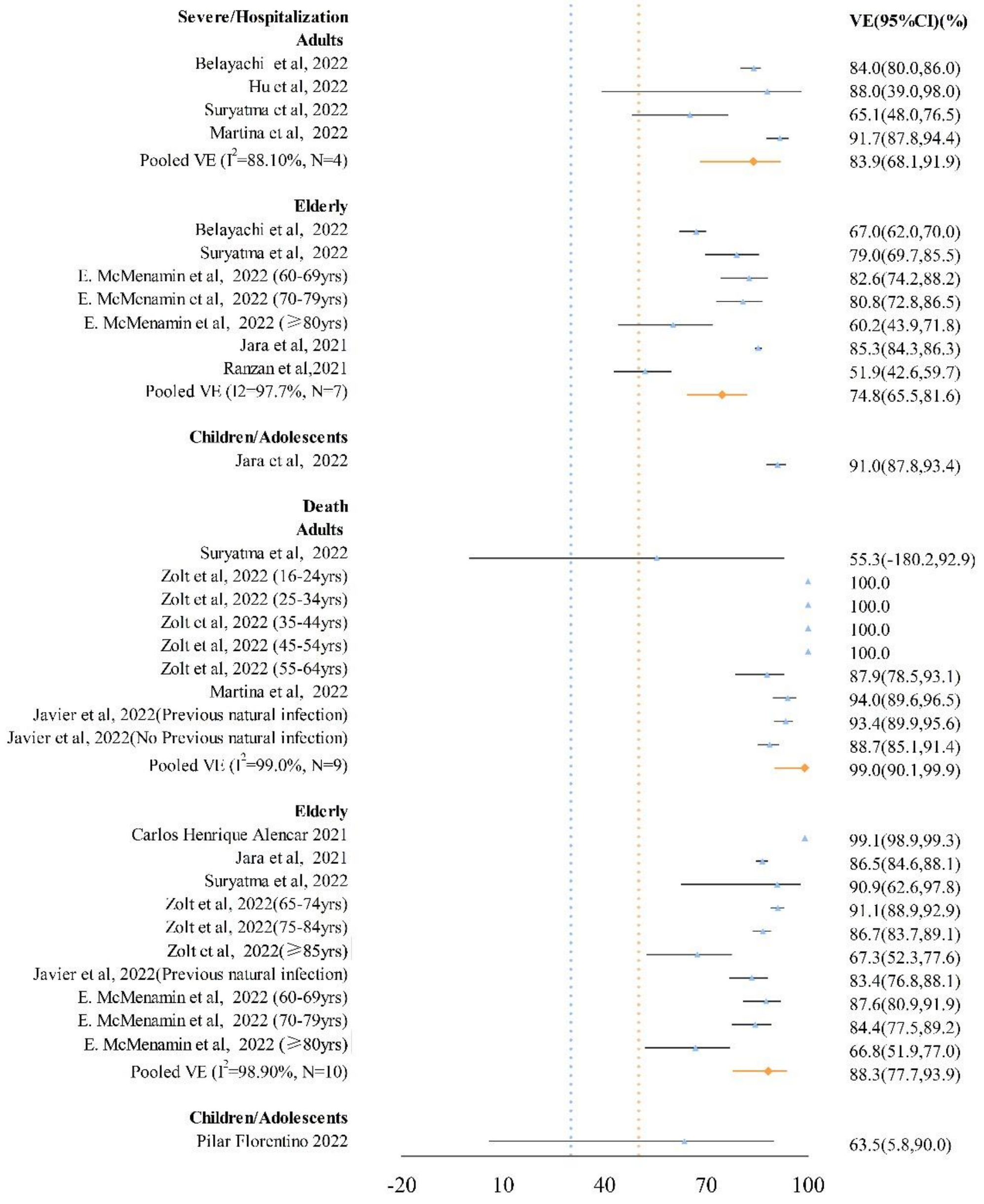

Supplement: Supplementary file 1 [file vaccines-12-00781-s001.zip › vaccines-3044314-supplementary.pdf]
